# Unlocking the Hidden Value of *Lycoris radiata* Leaves: Superior Bioactivities and Phytochemical Richness via Vacuum Ultrasonic-Assisted Extraction

**DOI:** 10.3390/plants15213384

**Published:** 2026-11-01

**Authors:** Guey-Horng Wang, Jong-Tar Kuo, Yi-Hsiang Lee, Ying-Chien Chung

**Affiliations:** 1 Research Center of Natural Cosmeceuticals Engineering, Xiamen Medical College, Xiamen 361008, China; 2 Department of Nutrition and Health Sciences, Kainan University, Taoyuan City 33857, Taiwan; 3 Department of Biological Science and Technology, China University of Science and Technology, Taipei City 115311, Taiwan

**Keywords:** cosmeceuticals, cytotoxicity, extraction, pharmacological properties, polyphenols, wound healing

## Abstract

*Lycoris radiata* is a medicinal and ornamental plant rich in Amaryllidaceae alkaloids and polyphenols. However, most studies have focused solely on its bulb extracts, neglecting the potential benefits of its leaves and roots. Due to the complex structures of its alkaloids and the difficulty of chemical synthesis, effective extraction methods are essential. This study evaluated the biological activities of different *L. radiata* tissues using a sequential experimental approach. Initially, leaves, bulbs, and roots were extracted via maceration, ultrasonic-assisted extraction (UAE), and vacuum ultrasonic-assisted extraction (VUAE) to compare their antioxidant properties (DPPH, ABTS, total phenolics, and total flavonoids). Based on superior yield and antioxidant capacity, VUAE was selected to prepare extracts from the three plant parts for comparative evaluations of cytotoxicity, antimicrobial activity, anti-inflammatory activity, and specific enzymatic inhibitions (tyrosinase, MMP-1, elastase, collagenase, and hyaluronidase). Furthermore, the leaf extract obtained via VUAE—which demonstrated prominent multifunctional efficacy—was exclusively analyzed for its phytochemical profile (via HPLC-UV/ESI-MS/MS), molecular docking binding affinities, in vitro wound healing capacity, and transdermal permeation efficiency. VUAE achieved the highest extraction yields across all tissues. VUAE-derived leaf extracts exhibited the strongest ABTS radical scavenging activity, notable skin-whitening effects, significant anti-inflammatory properties, and effective wound healing. Both root and leaf VUAE extracts showed potent anti-aging activity by inhibiting key skin-degrading enzymes, whereas bulb extracts demonstrated the most pronounced antimicrobial activity. All extracts maintained cell viability above 70%, and leaf extracts promoted wound healing in HaCaT and CCD966SK cells. Chemical profiling identified 31 alkaloids and 18 polyphenols in the VUAE leaf extracts, with lycorine, galanthamine, rutin, quercetin, and cinnamic acid as key components contributing to the observed multifunctional effects. In summary, VUAE-derived *L. radiata* extracts (particularly those from leaves) exhibit broad bioactivities, safety, and high extractability, highlighting their strong potential for applications in cosmetics, dermatology, and agricultural biotechnology.

## 1. Introduction

*Lycoris radiata*, commonly known as the red spider lily or stone garlic, is a bulbous plant in the Amaryllidaceae family, renowned for its distinctive flower shape and bright colors [1]. It is distributed across tropical and warm regions worldwide—including China, Korea, Japan, Nepal, Malaysia, Vietnam, and Taiwan—and typically thrives in shaded, moist habitats such as hillsides, forest edges, grasslands, and riverbanks. In Taiwan, its natural distribution is primarily limited to the outlying islands of Matsu and Kinmen. Valued for both its ornamental appeal and rich medicinal history, the entire *L. radiata* plant possesses therapeutic potential, with its bulbs traditionally serving as the primary source of bioactive pharmacological extracts [2,3,4].

The medicinal efficacy of *L. radiata* is primarily attributed to its high and diverse content of family-specific alkaloids, which serve as nitrogenous secondary metabolites. Amaryllidaceae plants encompass 18 primary structural classes [5], with *L. radiata* exhibiting the greatest structural variety among studied species [6]. These alkaloids vary in type and quantity across different plant tissues [7] and are mainly categorized into six groups: crinine-, galanthamine-, lycorine-, homolycorine-, tazettine-, and narciclasine-type alkaloids [8]. Among the specific compounds identified, galanthamine and lycoramine act as acetylcholinesterase inhibitors; notably, galanthamine has received FDA (Food and Drug Administration) approval for the treatment of Alzheimer’s disease [1,9]. Lycorine demonstrates broad biological properties, including antiviral, antibacterial, antimalarial, anti-inflammatory, and apoptotic activities against various cancer cell lines [10,11]. Other constituent alkaloids contribute additional antitumor, cytotoxic, neuroprotective, and wound-healing benefits [1,12]. Because these compounds possess highly intricate chemical structures, their large-scale commercial production via total chemical synthesis remains a major bottleneck, rendering direct extraction from *L. radiata* the most economically viable approach for industrial production [1,13].

Despite the high value of these alkaloids, conventional exploration of *L. radiata* faces two critical limitations: a narrow tissue focus and suboptimal extraction efficiency. Historically, research has predominantly concentrated on extracting compounds from the bulb, largely overlooking the potential pharmacological properties of other tissues, such as leaves and roots, which may also contain valuable bioactive compounds like polyphenols [1]. Furthermore, traditional extraction methods rely heavily on organic solvents—such as 70% to 90% ethanol, methanol, or trifluoroacetic acid solutions—followed by multi-step partitioning or chromatographic purification [2,7,14,15]. Although some recent studies have employed ultrasonic-assisted extraction (UAE) to enhance yield [16,17,18], conventional techniques still achieve low extraction efficiencies, ranging from 3.3% to 5.89% [19,20,21]. While these standard methods suffice for limited medical profiling of bulb extracts, developing optimized, sustainable extraction protocols is essential to unlock the potential of other plant parts for broader applications in cosmeceuticals and commercial health products.

To overcome efficiency and degradation challenges, vacuum ultrasonic-assisted extraction (VUAE) technology offers a promising alternative by combining ultrasonic cavitation with a negative-pressure vacuum environment [22,23]. Deaerating the solvent and extraction environment enhances cavitation, enabling deeper solvent penetration into the micropores and blind pores of plant tissue, thereby maximizing mass transfer and recovery of bioactive compounds [24]. Importantly, the vacuum environment lowers the solvent’s boiling point, which simultaneously accelerates extraction time, prevents thermal degradation of thermolabile compounds, and protects oxygen-sensitive phytochemicals from oxidation [22]. In this study, we systematically apply and compare maceration, UAE, and VUAE techniques using 80% ethanol to extract bioactive compounds from the leaves, bulbs, and roots of *L. radiata*. By comprehensively evaluating the pharmacological profiles and cytotoxicities of these extracts, this study aims to maximize the industrial and economic potential of the entire *L. radiata* plant.

## 2. Materials and Methods

### 2.1. Plant Material and Extraction Procedure

The plant was obtained from the Plant Breeding Facility located in Houli District, Taichung City, Taiwan. To ensure optimal tissue quality, different plant parts were harvested during their respective peak physiological activity periods: roots in September, leaves in January, and bulbs in March. The species was formally identified as *Lycoris radiata* by Professor Bau-Yuan Hu from the China University of Science and Technology (CUST). Voucher specimens (number 20201101-110) are preserved in the herbarium at CUST. After being washed with distilled water, the roots, bulbs, and leaves were separated and dried in an oven at 50 °C for 8 h. The dried materials were then ground using a high-speed grinder for 3 min. The resulting powder was passed through a 0.5 mm mesh and stored in a desiccator for later use.

Three extraction methods—maceration, UAE, and VUAE—were applied to process the roots, bulbs, and leaves of *L. radiata*. To preserve heat-sensitive polyphenols, the extraction temperature was maintained at 40 °C. For maceration, 2.5 kg of dried powder was soaked in 50 L of 80% ethanol at a 20:1 liquid-to-solid ratio, followed by agitation at 150 rpm for 3 h. UAE was performed using a PS-40AL ultrasonic machine (10 L capacity, Kejie Ultrasonic Technology Co., Shenzhen, China) with 5 L of 80% ethanol at 40 °C, applying sonication for 30 min at a 20:1 liquid-to-solid ratio, 240 W power, and 40 kHz frequency. VUAE employed a modified PS-40AL machine, enhanced with a silicone sealing ring around the stainless-steel cover to improve airtightness. A KF-40 vacuum adapter was installed at the center of the cover and connected via a vacuum rubber hose to a VP-20Pro vacuum pump (Joan Lab Equipment Co., Huzhou, China), maintaining a vacuum of −0.05 MPa. The extraction parameters matched those of UAE: 5 L of 80% ethanol, 40 °C, 30 min, 20:1 liquid-to-solid ratio, 240 W, and 40 kHz frequency. The extract obtained through VUAE was designated as the VUAE extract. After extraction by the different methods, the mixtures were filtered, and the filtrates were concentrated under vacuum at 40 °C before undergoing freeze-drying to produce the final product.

### 2.2. Microbial Strains, Cells, and Reagents

For the analysis of antimicrobial activity across various VUAE extracts, bacterial strains including *Escherichia coli* (ATCC 8739), *Pseudomonas aeruginosa* (ATCC 9027), *Staphylococcus aureus* (ATCC 6538), and *Cutibacterium acnes* (ATCC 6919), alongside fungal strains *Candida albicans* (ATCC 10231) and *Aspergillus brasiliensis* (ATCC 16404), were utilized. All microbial strains were acquired from the Bioresource Collection and Research Center (BCRC) in Hsinchu, Taiwan. Bacterial cultures were grown using tryptic soy broth (TSB) and tryptic soy agar (TSA), whereas fungal cultures were maintained in Sabouraud dextrose broth (SDB) and Sabouraud dextrose agar (SDA). The cell lines HaCaT, CCD966SK, and RAW 264.7 were also sourced from the BCRC. Human epidermal melanocytes (HEMn) were obtained from Cascade Biologics (Portland, OR, USA). HaCaT cells were cultured in Dulbecco’s Modified Eagle Medium supplemented with 10% fetal bovine serum (FBS) and 1% penicillin-streptomycin (P/S). CCD966SK cells were grown in Minimum Essential Medium supplemented with 10% FBS and 1% P/S (modified MEM), while RAW264.7 cells were maintained in RPMI 1640 medium supplemented with 10% FBS and 1% P/S (modified RPMI). HEMn cells were cultured in Medium 254 containing Human Melanocyte Growth Supplement (HMGS). Chemicals with a purity of 99% or higher and enzymes were purchased from Sigma-Aldrich (St. Louis, MO, USA), unless specified otherwise.

### 2.3. Assessment of Antioxidant Properties of Various Extracts Obtained Through Maceration, UAE, and VUAE

DPPH (2,2-diphenyl-1-picrylhydrazyl) radical scavenging activity of the extracts was evaluated using the method described by Wu et al. (2018) [25]. Extracts at various concentrations were mixed with a 0.25 mM DPPH solution in a 4:1 ratio, incubated in the dark at room temperature for 30 min, and the absorbance was measured at 517 nm using a UV–visible spectrophotometer (UV-2600i, Shimadzu, Kyoto, Japan). The scavenging activity (%) was calculated as follows:

(1) $$\mathrm{DPPH scavenging activity}\left(\%\right)=\left[\frac{A_{\mathrm{control}}-A_{\mathrm{sample}}}{A_{\mathrm{sample}}}\right]\times 100$$

A_control_: Absorbance of the DPPH solution with the solvent only
A_sample_: Absorbance of the DPPH solution with the extract

The antioxidant potency was expressed as the IC_50_ value, representing the concentration required to inhibit 50% of the DPPH radicals.

ABTS [2,2′-azino-bis(3-ethylbenzothiazoline-6-sulfonic acid)] radical scavenging activity of the extracts was measured according to Wu et al. (2018) [25]. A 7 mM ABTS stock solution was combined with 2.45 mM potassium persulfate in a 1:1 ratio to generate the ABTS radical cation, which was allowed to develop in the dark at room temperature for 16 h. Prior to the assay, this dark-colored solution was diluted with ethanol to achieve an initial absorbance of 0.7 ± 0.02 at 734 nm. For the assay, 20 μL of extract at various concentrations was mixed with 180 μL of the diluted ABTS solution, incubated for 30 min protected from light, and absorbance was measured at 734 nm using an ELISA plate reader (BioTek Instruments, Santa Clara, CA, USA). The percentage of radical scavenging was calculated as follows:

(2) $$\mathrm{ABTS scavenging activity}\left(\%\right)=\left[\frac{A_{\mathrm{control}}-A_{\mathrm{sample}}}{A_{\mathrm{sample}}}\right]\times 100$$

A_control_: Absorbance of the ABTS solution with the solvent blank
A_sample_: Absorbance of the ABTS solution with the extract

Butylated hydroxytoluene (BHT) served as a positive control in both the DPPH and ABTS radical scavenging assays.

The total phenolic content (TPC) of the samples was determined using the Folin–Ciocalteu colorimetric method as described by Chen et al. (2025) [26]. Briefly, 0.5 mL of either the standard solution or extract was mixed with 1 mL of 1 N Folin–Ciocalteu reagent and allowed to react for 5 min to facilitate the oxidation of phenolic compounds. Subsequently, 1 mL of a 7.5% sodium carbonate solution was added, and the reaction mixture was incubated in the dark at 28 °C for 3 h. The absorbance of the supernatant was measured at 760 nm using a UV–visible spectrophotometer (Thermo Fisher Scientific, Waltham, MA, USA). TPC values were calculated using a gallic acid standard calibration curve and expressed as milligrams of gallic acid equivalents per gram of dry weight (mg GAE/g dw). Total flavonoid content (TFC) was measured using the aluminum chloride colorimetric assay following the protocol described by Chen et al. (2025) [26]. In summary, 0.5 mL of either the standard solution or extract was mixed with 0.5 mL of a 2% aluminum chloride solution and incubated at 28 °C for 60 min. The absorbance of the supernatant was then recorded at 420 nm using a UV–visible spectrophotometer. TFC values were calculated using a rutin standard curve and expressed as milligrams of rutin equivalents per gram of dry weight (mg RE/g dw).

### 2.4. Measurement of Tyrosinase Activity and Cellular Melanin Content

Extracellular tyrosinase inhibition was assessed using a modified version of the method described by Wu et al. (2018) [25]. The assay involved mixing 50 μL of VUAE extract (dissolved in DMSO) with 50 μL of mushroom tyrosinase (350 units in 0.05 M phosphate buffer, pH 6.8) and pre-incubating the mixture at 37 °C for 10 min to allow potential interactions between the inhibitor and the enzyme. Then, 50 μL of a 100 mg/L tyrosine solution was added to initiate the enzymatic reaction. After 20 min, the absorbance was measured at 490 nm using an ELISA plate reader (Epoch, Biotek, Santa Clara, CA, USA). Kojic acid or arbutin was used as a positive control, while a solution without extract served as a negative control. Tyrosinase inhibition (%) was calculated based on absorbance values: A (control with enzyme), B (control without enzyme), C (sample with enzyme), and D (sample without enzyme).

(3) $$\mathrm{Tyrosinase inhibition}\left(\%\right)=\frac{\left[(A-B)-(C-D)\right]}{\left(A-B\right)}\times 100$$

To assess the inhibitory effect on intracellular tyrosinase activity, a cell-based assay was performed using HEMn cells, following the methodology described by Wu et al. (2021) [27]. Briefly, HEMn cells were seeded into 24-well plates at a density of 6 × 10^5^ cells per well in Medium 254 supplemented with HMGS and incubated at 37 °C under 5% CO_2_ until reaching the desired confluence. Subsequently, the cells were treated with various concentrations of the VUAE extract for 24 h. After incubation, the cells were washed with ice-cold PBS, detached using trypsin, and lysed with lysis buffer. The resulting lysates were clarified by centrifugation at 10,000× *g* for 20 min at 4 °C. Tyrosinase activity was measured by mixing the protein lysate with a 2.5 mM L-DOPA solution, followed by incubation at 37 °C for 1 h. The absorbance of the produced dopachrome was then measured at 475 nm using an ELISA plate reader.

HEMn cells were cultured and treated as previously described. The cells were detached and then centrifuged to form a pellet. To dissolve the melanin, the pellet was resuspended in 1 M NaOH containing 10% DMSO and heated at 80 °C for 1 h. After cooling, the absorbance of the supernatant was measured at 450 nm using an ELISA plate reader [25]. Melanin levels were normalized to the total protein content in the cell lysate, which was determined by the Bradford protein assay. The results were expressed as a percentage relative to the control group. Synthetic melanin was used as a standard for calibration and absolute quantification.

### 2.5. Assessment of the Anti-Inflammatory Activity of VUAE Extract

The anti-inflammatory effects of the VUAE extract were evaluated by measuring levels of nitric oxide (NO), interleukin-6 (IL-6), and tumor necrosis factor-alpha (TNF-α), as well as the activities of cyclooxygenase-1 (COX-1) and cyclooxygenase-2 (COX-2) [28]. RAW 264.7 cells were plated at 5 × 10^5^ cells/well and incubated for 24 h at 37 °C with 5% CO_2_. The cells were pre-treated with the VUAE extract for 2 h, followed by stimulation with 1 mg/L of lipopolysaccharide for an additional 24 h. NO levels were determined using the Griess reagent, while TNF-α and IL-6 concentrations were measured using Quantikine ELISA kits (R&D Systems, Minneapolis, MN, USA) [29]. The extracellular activities of COX-1 and COX-2 were assessed using COX (human) Inhibitor Screening Assay kits (Cayman Chemical, Ann Arbor, MI, USA) [28]. Indomethacin was used as a positive control in all assays.

### 2.6. Assessment of the Antiaging Activity of VUAE Extract

The inhibitory effect on MMP-1 (matrix metalloproteinase-1) activity was assessed using CCD966SK cells. These cells were cultured in modified MEM at 37 °C in a humidified incubator with 5% CO_2_. For the assay, cells were seeded at a density of 3 × 10^5^ cell per well in 24-well plates and incubated for 24 h. After removing the culture medium, 200 μL of VUAE extract at various concentrations was added, and the cells were incubated for an additional 48 h. The culture supernatant was then collected and centrifuged at 2000× *g* for 10 min at 4 °C. MMP-1 levels in the supernatant were measured using a Human MMP-1 ELISA Kit from RayBiotech (Norcross, GA, USA), following the manufacturer’s protocol [30].

Specific methods were employed to measure the extracellular activities of collagenase, elastase, and hyaluronidase. Collagenase activity was assessed using the fluorescent dye-quenched gelatin method [30]. For elastase activity, 20 μL of VUAE extract, 50 μL of porcine pancreatic elastase at 1 unit/mL, and 5 μL of 0.8 mM Suc-Ala-Ala-Ala-pNA in Tris-HCl buffer (pH 8.0) were mixed and incubated at 25 °C for 20 min; absorbance was then recorded at 410 nm [31]. Hyaluronidase activity was evaluated using a slightly modified method from Thring et al. (2009) [31]: 100 μL each of VUAE extract and bovine testis hyaluronidase at 1 unit/mL, along with 500 μL of hyaluronic acid solution in 0.1 M acetate buffer (pH 3.5), were combined and incubated at 35 °C for 40 min. The N-acetylglucosamine produced was reacted with 2 mL of *p*-DMAB, and absorbance was measured at 570 nm. In all assays, epigallocatechin gallate (EGCG) was used as a positive control, and inhibition percentages were calculated relative to the untreated control.

### 2.7. Assessment of the Antimicrobial Activity of VUAE Extract

The antimicrobial activity of the VUAE extract was evaluated by determining the minimum bactericidal concentration (MBC) for *E. coli*, *P. aeruginosa*, *S. aureus*, and *C. acnes*, as well as the minimum fungicidal concentration (MFC) for *C. albicans* and *A. brasiliensis*. Bacterial cultures were grown at 32.5 °C in tryptic soy broth (TSB), while fungal cultures were incubated at 22.5 °C in Sabouraud dextrose broth (SDB). After incubation, mixtures containing 1 mL of VUAE extract at various concentrations, 1 mL of bacterial or spore suspension (8.0 × 10^5^ CFU/mL or spores/mL), and 8 mL of fresh broth were prepared and incubated at 32.5 °C for 24 h for bacteria or at 22.5 °C for 7 days for fungi. Samples were then plated on tryptic soy agar (TSA) and Sabouraud dextrose agar (SDA), and cell counts were determined using serial dilution methods. The MBC was defined as the lowest concentration of the extract that killed 99.9% of the bacteria, while the MFC was the lowest concentration that completely inhibited fungal growth. Streptomycin was used as a positive control for *E. coli*, *S. aureus*, and *P. aeruginosa*; erythromycin served as the positive control for *C. acnes*; and nystatin was the positive control for *C. albicans* and *A. brasiliensis*.

### 2.8. Assessment of Cell Viability and Wound Healing Capacity of VUAE Leaf Extract

The effect of VUAE extract on the viability of HaCaT, CCD966SK, HEMn, and Raw 264.7 cells was evaluated using the Cell Counting Kit-8 (Abbkine Scientific Co., Atlanta, GA, USA) according to the manufacturer’s instructions. Each cell line was seeded at an initial density of 5 × 10^3^ cells/well in 100 μL of its respective culture medium and incubated for 24 h at 37 °C in a humidified atmosphere containing 5% CO_2_. The medium was then replaced with 100 μL of fresh medium containing various concentrations of VUAE extract, and incubation continued for an additional 72 h. Control wells were treated with medium alone. After the treatment period, 10 μL of CCK-8 reagent was added to each well, followed by a 1 h incubation at 37 °C. Finally, the absorbance at 450 nm was measured for each well.

For the in vitro wound healing assay, the method described by Bazzicalupo et al. (2021) was followed [32]. HaCaT and CCD966SK cells were seeded into 6-well plates at a density of 5 × 10^5^ cells/well. Once the cells reached 90–100% confluence, forming a uniform monolayer, a straight scratch was made across the monolayer using a sterile 200 μL plastic pipette tip held at a consistent 45° angle. The resulting debris was removed by gently washing the wells twice with pre-warmed PBS. Subsequently, the cells were treated with 2 mL of fresh medium containing varying concentrations of VUAE leaf extract (0–130 mg/L). The negative control group received medium without extract. After incubation, the cells were fixed with FineFIX and stained with toluidine blue O. Wound closure percentage in HaCaT cells and cell density within the scratched area for CCD966SK cells were evaluated at 0 and 24 h post-scratch using a Nikon SMZ445 MP stereomicroscope (Nikon Instruments Inc., Tokyo, Japan) and quantified with ImageJ software (version 1.51p). Allantoin served as the positive control in the wound healing assay.

### 2.9. Analysis of Chemical Composition in VUAE Leaf Extract

The method for extracting Amaryllidaceae alkaloids (AAs) was adapted from the protocol by Tian et al. (2015) with minor modifications [16]. Lyophilized VUAE leaf extract was dissolved in 5% hydrochloric acid and then extracted with petroleum ether. The pH of the aqueous phase was adjusted to 9.5 using ammonia and re-extracted with chloroform. The chloroform layer, containing crude AAs, was concentrated using an Oasis MCX column (Waters Co., Milford, MA, USA) according to the manufacturer’s instructions. After drying with a high-pressure purge concentrator (EYELA, Taipei, Taiwan), the sample was re-dissolved in 5% methanol, filtered through a 0.22 μm filter, and prepared for HPLC-UV/ESI-MS/MS analysis.

A Hitachi HPLC Chromaster system (Hitachi, Tokyo, Japan), consisting of an HPLC pump (model 5160), autosampler (model 5260), column oven (model 5310), UV-Vis detector (model 5420), and MS detector (model 5610), was used to analyze AAs. Separation was performed using a Prodigy ODS-3 column (Phenomenex, Torrance, CA, USA) (2.0 mm × 150 mm, 5 µm). The mobile phases were 40 mM ammonium acetate aqueous solution (A) and acetonitrile (B). The optimized gradient elution program was as follows: 0–15 min at 5% B; 15–17 min from 5% to 10% B; 17–20 min at 10% B; 20–30 min from 10% to 20% B; and 30–60 min from 20% to 70% B. Samples (20 µL) were injected at a flow rate of 0.3 mL/min, with the column temperature maintained at 30 °C. Detection was carried out at a wavelength of 232 nm. Mass spectrometry parameters included a scan range of 150–1000 *m/z*, spray voltage of 3 kV, cone voltage of 40 V, vaporizer temperature at 350 °C, capillary temperature at 250 °C, sheath gas pressure of 40 psi, and auxiliary gas pressure of 10 psi.

For the analysis of polyphenolic compounds, the lyophilized VUAE leaf extract was dissolved in 1 mL of 70% aqueous methanol and loaded onto a preconditioned minicolumn. After rinsing the column with 2 mL of distilled water, the extract was eluted twice with 2 mL of 90% methanol. The resulting purified extract was evaporated to dryness and then reconstituted in a 1:1 (*v/v*) water/methanol mixture for HPLC analysis. The same Hitachi HPLC Chromaster system was used. Separation was performed using a Prontosil C18 column (Bischoff, Leonberg, Germany) (250 mm × 4.0 mm, 5 μm). The mobile phase consisted of solvent A (0.025% trifluoroacetic acid in water) and solvent B (acetonitrile). The elution method and operating conditions followed the protocol described by Lin et al. (2024) [33]. LC peaks were identified via LC-MS/MS and compared with reference standards or literature data. The relative amounts (%) of AAs and polyphenolic compounds were calculated based on the peak areas obtained from the LC analysis.

### 2.10. Analyses of Transdermal Absorption and Permeation

This study utilized a Franz-type vertical diffusion cell, as described by Wang et al. (2023) [34], to evaluate the skin absorption of VUAE leaf extract at concentrations of 400 mg/L and 80 mg/L. These concentrations were selected based on their potential whitening, anti-inflammatory, anti-wrinkle, antibacterial, and wound-healing properties. Additionally, a lotion formulation containing 84.96–84.992% distilled water, 12% jojoba oil, 3% Emulsifying Wax NF, and 0.008–0.04% VUAE leaf extract was tested to examine the influence of the formulation matrix on absorption. For each assay, 1 mL of the sample and 6 mL of 0.9% sodium chloride solution were placed in the donor and receptor chambers, respectively, separated by a Strat-M membrane with an area of 1.77 cm^2^ (Merck Millipore, Billerica, MA, USA). The diffusion cells were maintained at 32 °C with continuous stirring at 500 rpm. Hourly samples were collected and replaced with fresh saline until the TPC in the receptor chamber reached a steady state.

### 2.11. Statistical Analysis

The data are expressed as means ± standard deviations (SD). Statistical analyses were performed using SPSS software (version 26; IBM Corp., Armonk, NY, USA). Differences among experimental groups were evaluated by one-way analysis of variance (ANOVA). When significant differences were found, Duncan’s multiple range test was applied for post hoc multiple comparisons to identify specific treatment effects. Statistical significance was defined as *p* < 0.05. The IC_50_ values were calculated using GraphPad Prism software (version 9; GraphPad Software, San Diego, CA, USA).

## 3. Results and Discussion

### 3.1. Antioxidant Properties of L. radiata Extracts Obtained by Different Methods

To assess the effectiveness of three extraction techniques for obtaining compounds from *L. radiata*, the same extraction solvent, liquid-to-solid ratio, and extraction temperature were used to further compare the performance of UAE and VUAE. The extraction temperature was maintained at 40 °C to prevent the degradation of heat-sensitive polyphenols. The results showed that, based on lyophilized extracts, the extraction yields from different parts of *L. radiata* ranged from 3.96% to 5.62% for maceration, 4.73% to 7.16% for UAE, and 5.92% to 9.35% for VUAE. Among these techniques, VUAE achieved the highest extraction yield, whereas maceration produced the lowest. Additionally, UAE was at least 1.2 times more effective than maceration, and VUAE was at least 1.5 times more effective. Furthermore, all methods yielded the most from leaves, followed by bulbs and roots. In this study, the extraction yield of VUAE from *L. radiata* bulbs (7.06%) was notably higher than yields reported in earlier studies using maceration, which ranged from 1.17% to 5.89% [15,20,21].

**Figure 1:**
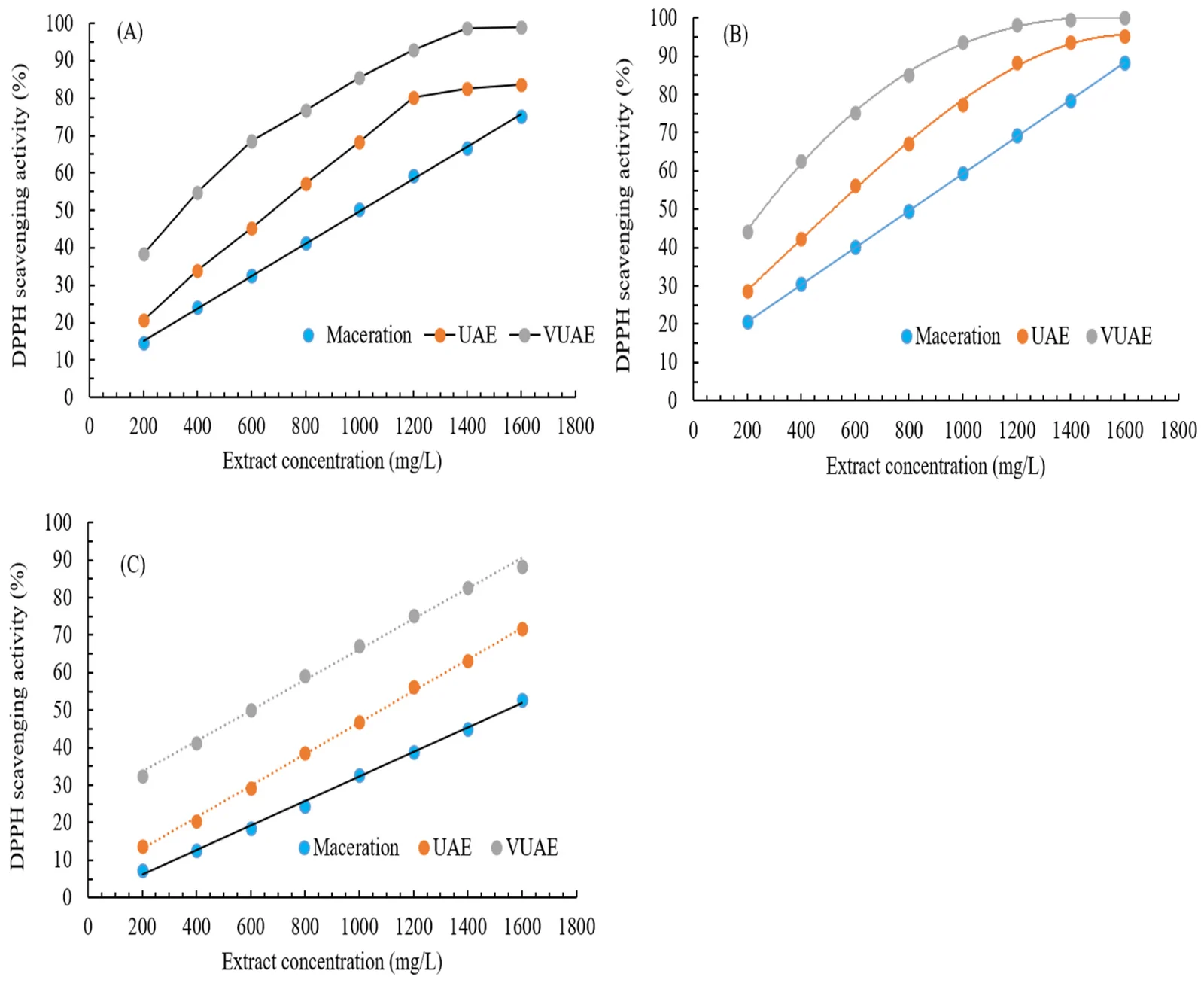
Effects of different extraction techniques on the DPPH scavenging activity of *L. radiata* leaf (**A**), bulb (**B**), and root (**C**) extracts. Extraction conditions for maceration: extraction solvent, 80% ethanol; liquid-to-solid (L/S) ratio, 20 mL/g; extraction temperature, 40 °C; and extraction time, 3 h. For UAE: extraction time, 30 min; and ultrasonic power, 240 W. For VUAE: vacuum pressure, −0.05 MPa. Data are presented as the means ± standard deviations of three independent experiments.

**Figure 2:**
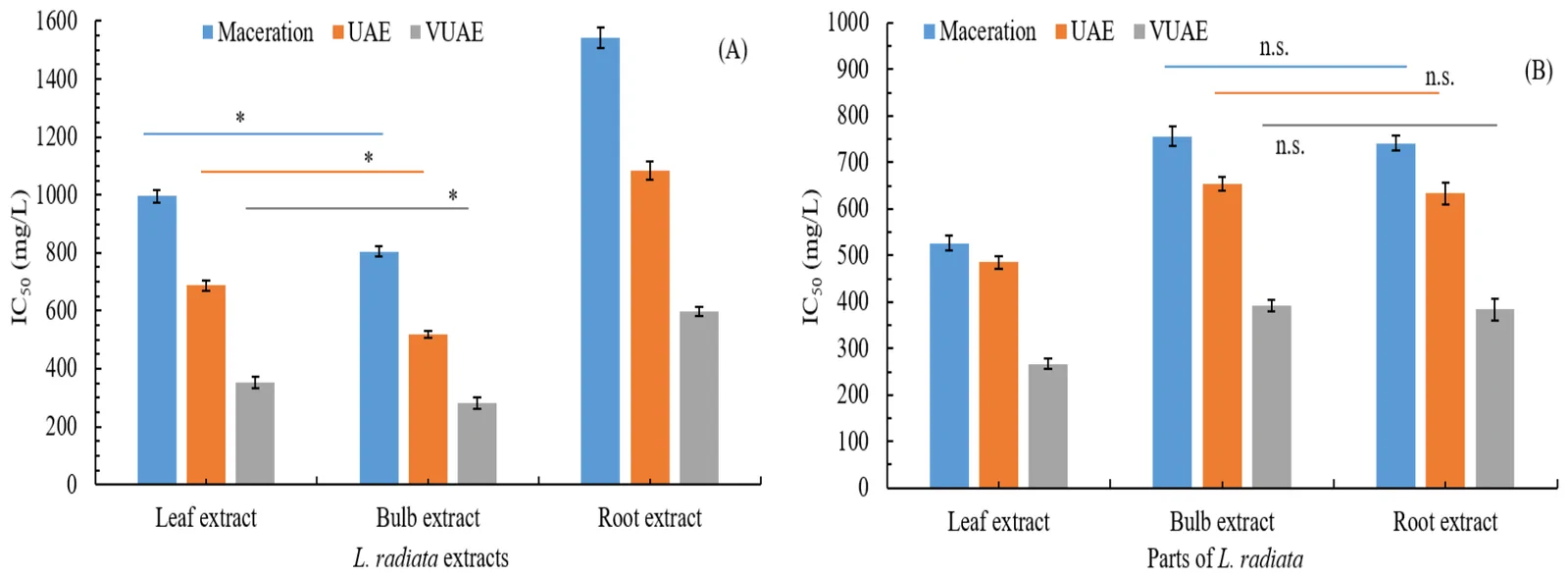
IC_50_ values for the DPPH scavenging activity (**A**) and ABTS scavenging activity (**B**) of *L. radiata* extracts obtained using different extraction techniques. Data are presented as the means ± standard deviations of three independent experiments. (ns, nonsignificant; *p* > 0.05; significance, * *p* < 0.05).

Figure 1 shows the impact of various extraction techniques on the DPPH radical scavenging activity of *L. radiata* extracts. The findings revealed that as extract concentration increased, the ability to scavenge DPPH radicals also rose. Extracts obtained through VUAE demonstrated greater effectiveness in scavenging DPPH radicals compared to those obtained by UAE or maceration. To further explore extracts from different parts of *L. radiata*, Figure 2 presents the IC_50_ values for each extract’s ability to neutralize DPPH and ABTS radicals. The data revealed that the bulb extract showed the strongest DPPH radical scavenging activity, followed by the leaf and then the root extracts. Conversely, for ABTS radical scavenging, the leaf extract was most effective, with the root and bulb extracts showing similar levels of activity (*p* > 0.05). These variations are likely due to differences in the hydrophilic or hydrophobic nature of the radicals and the concentrations of active compounds in each extract [35]. Specifically, the IC_50_ value for DPPH scavenging of the bulb extract prepared by VUAE was 281.4 ± 19.2 mg/L, whereas the leaf extract prepared by VUAE had an IC_50_ of 267.2 ± 10.8 mg/L for ABTS radical removal. Both were less potent than BHT, a compound with potential health risks, which had IC_50_ values of 53.4 ± 3.2 mg/L and 45.8 ± 2.7 mg/L for DPPH and ABTS scavenging, respectively.

Table 1 lists TPC and TFC levels in *L. radiata* extracts obtained through various extraction techniques. TPC correlates with DPPH scavenging activity, while TFC aligns with ABTS activity, probably due to the potent ABTS scavenging effects of flavonoids [35]. UAE boosts both TPC and TFC compared to maceration, with VUAE proving even more effective. These findings align with those reported by Huang et al. (2024) [23]. Specifically, in bulb extracts, UAE raises TPC by 30.7% over maceration, with VUAE adding another 18.6%. For leaf extracts, UAE increases TFC by 17.1%, and VUAE adds 23.5%. Overall, VUAE is more efficient at boosting TPC than TFC in the extracts. Although data on TPC and TFC content in *L. radiata* extracts are scarce, a similar trend has been observed in *Hymenocallis littoralis*, another member of the Amaryllidaceae family. In that species, TPC levels rank as bulb extract > leaf extract > root extract, while the TFC levels follow leaf extract > root extract ≥ bulb extract [35].

### 3.2. Whitening Properties of L. radiata Extracts Obtained Through VUAE

Previously, conventional *L. radiata* extracts had rarely been shown to inhibit tyrosinase activity, possibly because of factors such as environmental conditions, fertilization, and extraction methods. Most prior research has focused on preventing the proliferation of melanoma cells instead of aiming to inhibit tyrosinase [36].

The VUAE extracts yielded the highest amounts and exhibited strong antioxidant properties, making them the primary focus of further study. Among these, the leaf extract showed the strongest whitening effect, outperforming bulb and root extracts by inhibiting melanin production 1.5 times more than the bulb extract and 9.8 times more than the root extract (see Table 2). The whitening activity was found to be associated with the levels of phenolics and flavonoids present (Table 1). While the root extract consistently reduced tyrosinase activity and melanin production, the leaf and bulb extracts displayed varied inhibition patterns. Specifically, the leaf extract showed inconsistent inhibition of both intracellular and extracellular tyrosinase activity, with stronger inhibition of intracellular tyrosinase activity; however, there was no significant difference in the degree of inhibition between intracellular tyrosinase activity and melanin production (*p* > 0.05). The bulb extract demonstrated the most complex whitening mechanism, with irregular inhibition of intracellular and extracellular tyrosinase activity as well as melanin production. This complexity is attributed to the presence of diverse polyphenols and alkaloids in the bulb extracts, whereas the simpler polyphenol profile in leaf extracts contributed to more consistent intracellular whitening effects [37]. The VUAE leaf extract had an IC_50_ value of 93.5 ± 1.4 mg/L for extracellular tyrosinase inhibition, outperforming the positive control α-arbutin (125.8 ± 10.2 mg/L) but less potent than kojic acid (16.2 ± 1.7 mg/L). This study is the first to validate the whitening properties of *L. radiata*.

### 3.3. Anti-Inflammatory Activity of L. radiata Extracts Obtained Through VUAE

**Figure 3:**
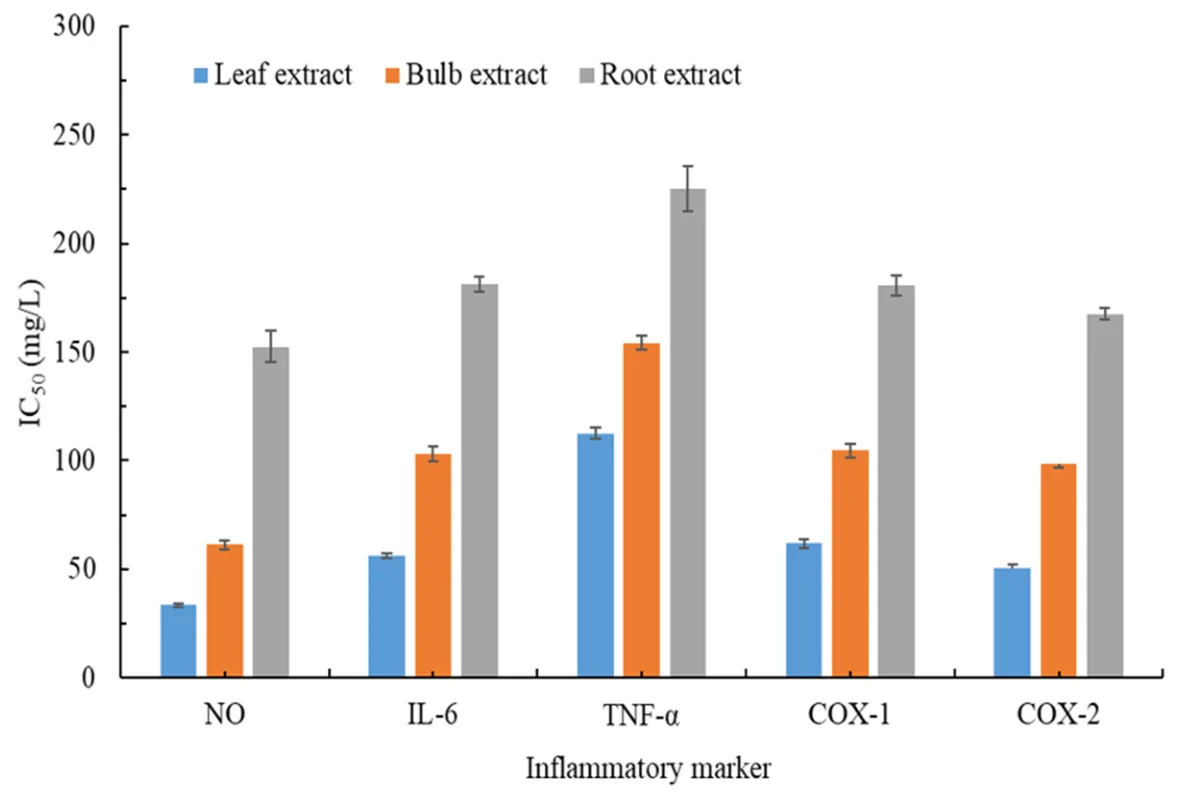
IC_50_ values for the anti-inflammatory activity of *L. radiata* extracts obtained through VUAE. Extraction conditions: extraction solvent, 80% ethanol; liquid-to-solid (L/S) ratio, 20 mL/g; extraction temperature, 40 °C; extraction time, 30 min; ultrasonic power, 240 W; and vacuum pressure, −0.05 MPa. Data are presented as the means ± standard deviations of three independent experiments.

Compounds isolated from *L. radiata*, such as narciclasine and lycorine, have demonstrated anti-inflammatory activity [26]. This research employs both cell-based and enzyme-based assays to evaluate the anti-inflammatory activity of *L. radiata* extracts, aiming to expand their potential uses. Among the extracts obtained via VUAE, the leaf extract was the most effective at reducing NO production in the cell-based assay, showing the strongest anti-inflammatory activity (IC_50_: 33.6 ± 0.8 mg/L to 112.7 ± 2.5 mg/L); the root extract showed the least effect (see Figure 3). In the enzyme-based assay, VUAE extracts most strongly inhibited COX-2 activity, with the leaf extract (IC_50_: 50.6 ± 1.4 mg/L to 61.8 ± 2.0 mg/L) outperforming the bulb and root extracts (see Figure 3). When compared to alkaloids such as (+)-*N*-methoxycarbonyl-2-demethylisocorydione and narciclasine, which were isolated and purified from *L. radiata* bulbs and have IC_50_ values of 33.87 mg/L for COX-1 and 0.003 mg/L for NO [15,21], the VUAE leaf extracts are less potent. Nevertheless, compared to the positive control indomethacin, which has IC_50_ values of 20.16 ± 3.1 mg/L for NO, 35.42 ± 5.2 mg/L for IL-6, 52.68 ± 7.5 mg/L for TNF-α, 57.02 ± 10.4 mg/L for COX-1, and 53.61 ± 9.5 mg/L for COX-2, the VUAE leaf extracts exhibit satisfactory efficacy, indicating their promising potential for use in cosmeceutical products.

### 3.4. Anti-Aging Activity of L. radiata Extracts Obtained Through VUAE

Extracts from *L. radiata* demonstrate antioxidant properties and thus have potential anti-aging benefits; however, current research focuses on their bioactive compounds and medicinal properties. This area remains underexplored, prompting us to conduct a more in-depth study of enzyme activities associated with anti-aging and anti-wrinkle effects. Table 3 shows the anti-aging activities of *L. radiata* extracts obtained through VUAE. Since the inhibition patterns differ among enzymes, IC_50_ values are typically used to assess their inhibitory potency. Overall, the anti-aging activity ranked as follows: root extracts ≥ leaf extracts > bulb extracts. Elastase was the enzyme most readily inhibited, followed by MMP-1, hyaluronidase, and collagenase. The order of inhibition may vary depending on the specific components present in the extracts [38]. By blocking these enzymes, the aging process can be slowed down, preserving the skin’s structural integrity and resulting in firmer, more elastic, and less wrinkled skin.

This finding slightly contrasts with the pattern observed in the antioxidant effects of *L. radiata* extracts. This discrepancy may be due to the fact that anti-aging activity is mainly linked to the type and amount of flavonols present in the extract [39]. Flavonols, such as quercetin, quercitrin, kaempferol, myricetin, catechin, luteolin, and apigenin, have been demonstrated to inhibit all four enzymes [40]. Most of these compounds are found in leaf extracts (see Table 6). The anti-aging effects of the root or leaf extracts surpassed those of the positive control EGCG (whose IC_50_ values against MMP-1, collagenase, elastase, and hyaluronidase were 90.6 ± 11.3 mg/L, 117.4 ± 12.8 mg/L, 95.7 ± 7.3 mg/L, and 426.7 ± 35.2 mg/L, respectively) in three anti-aging assays, suggesting that the *L. radiata* extracts obtained through VUAE exhibit potent anti-aging and anti-wrinkle properties.

### 3.5. Antimicrobial Activity of L. radiata Extracts Obtained Through VUAE

Lee et al. (2014) found that the antimicrobial properties of methanol-extracted bulb extracts from *L. radiata* might be attributed to phytosterols or alkaloids such as lycorenine and lycorine [41]. Additionally, leaves from plants in the Amaryllidaceae family contain various phenolic acids and flavonoids, which also exhibit antimicrobial effects [35]. Building on this, the current study assessed the antimicrobial activity of *L. radiata* extracts obtained through VUAE (Table 4). The findings revealed that the bulb extract demonstrated the strongest bactericidal activity (MBC ranging from 108 ± 11.8 mg/L to 150 ± 40.8 mg/L), followed by the leaf extract, while the root extract was considerably less effective. Similarly, the bulb extract showed the most potent fungicidal activity (MFC between 158 ± 11.8 mg/L and 225 ± 20.4 mg/L), whereas the root extract had almost no antifungal activity (MFC > 2000 mg/L). For comparison, the positive control streptomycin had MBC values ranging from 50 ± 8.2 mg/L to 125 ± 20.4 mg/L against *E. coli*, *S. aureus*, and *P. aeruginosa*. Erythromycin’s MBC against *C. acnes* was 25 ± 4.1 mg/L, and nystatin’s MFC against *C. albicans* and *A. brasiliensis* ranged from 80 ± 16.3 mg/L to 147 ± 9.4 mg/L. These findings suggest that *L. radiata* bulb extracts obtained through VUAE possess broad-spectrum antimicrobial activity, highlighting their potential for industrial use.

### 3.6. Cytotoxicity of L. radiata Extracts Obtained Through VUAE

**Figure 4:**
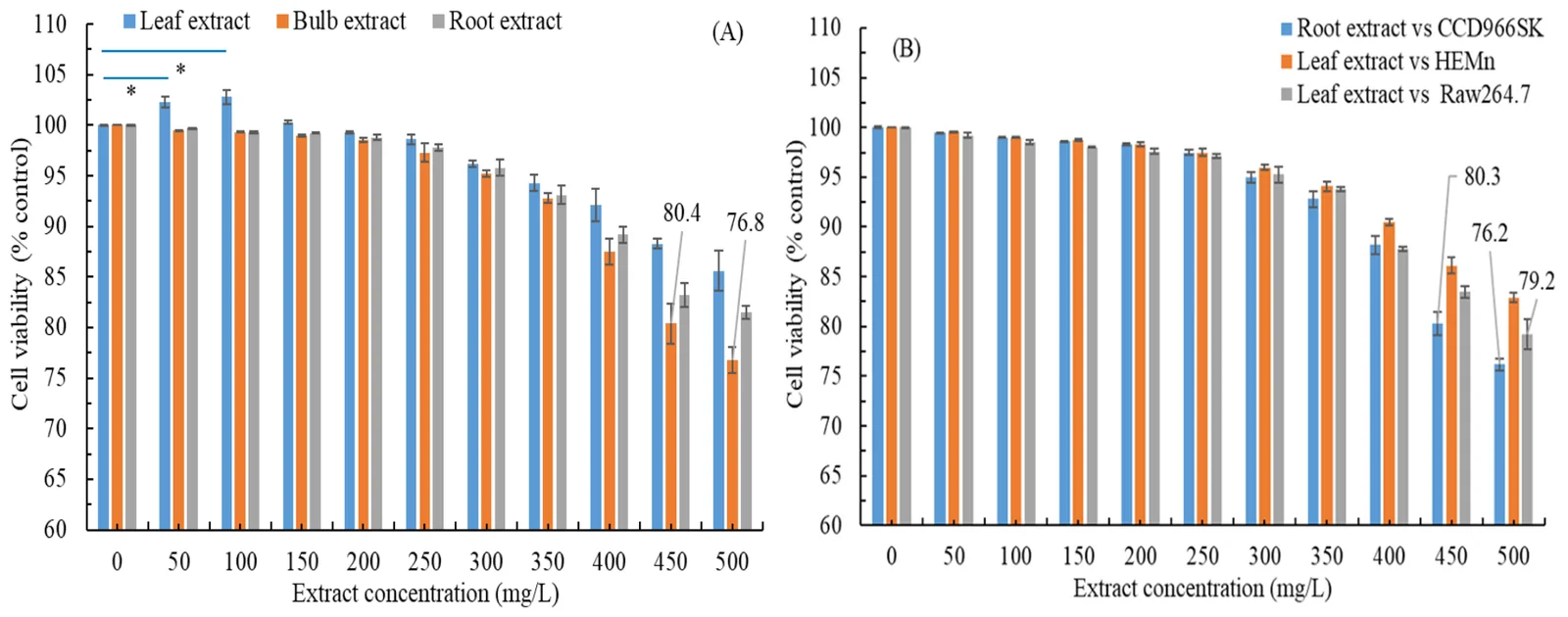
(**A**) Cytotoxic effects of *L. radiata* extracts obtained through VUAE on HaCaT cells. (**B**) Cytotoxic effects of *L. radiata* extracts obtained through VUAE on CCD966SK, HEMn, and Raw 264.7 cells. Data are presented as the means ± standard deviations of three independent experiments. (Significance, * *p* < 0.05).

Previous sections have demonstrated that *L. radiata* extracts obtained through VUAE possess a wide range of pharmacological activities. However, cytotoxicity testing is crucial to determine safe dosage levels and assess their suitability for safe development and use. Certain compounds isolated from *L. radiata*, like lycorine and narciclasine, are known to be toxic [26]. If VUAE extracts prove non-toxic at effective doses, their potential for industrial use will likely increase. Regardless of the intended target cells for these medicinal compounds, it is important to assess their impact on the outermost HaCaT skin cells upon application. As illustrated in Figure 4A, higher extract concentrations result in greater cytotoxicity. The bulb extract showed the greatest cytotoxicity, reducing cell viability to 76.8 ± 1.3% at 500 mg/L. Conversely, the leaf extract exhibited the least cytotoxicity and even significantly promoted cell growth at low concentrations (50–100 mg/L) (*p* < 0.05). According to the ISO 10993-5 standard [42], cell viability above 70% is considered non-toxic; thus, all tested extracts are deemed safe at the concentrations studied.

Building on our earlier studies, the leaf extract showed notable whitening and anti-inflammatory properties; therefore, we tested its cytotoxic effects on HEMn and Raw 264.7 cells. Since the root extract showed considerable anti-aging activity, we evaluated its cytotoxicity on CCD966SK cells, which are common pharmacological targets of these extracts. As illustrated in Figure 4B, cytotoxicity increased with higher extract concentrations. The root extract exhibited the strongest cytotoxicity against CCD966SK cells, reducing cell viability to 76.2 ± 0.5% at 500 mg/L. The leaf extract was more toxic to Raw 264.7 cells than to HEMn cells. Nevertheless, all extracts maintained cell viability above 70%, indicating no significant cytotoxicity and confirming their safety at the tested doses. Further analysis showed that the leaf extract was most cytotoxic to Raw 264.7 cells, followed by HEMn cells, and least cytotoxic to HaCaT cells. Since RAW 264.7 cells are immune cells, they are susceptible to oxidative stress and prone to damage or apoptosis [43]. In contrast, HaCaT cells possess strong structural stability and high tolerance [44]. Literature reports that narciclasine, isolated and purified from *L. radiata*, selectively induces apoptosis in cancer cells without affecting normal fibroblasts [45]. Shen et al. (2019) found that narciclasine significantly inhibits the proliferation of Raw 264.7 cells [21]. The effects of alkaloids isolated from *L. radiata* on different cancer cells also vary [3], with some alkaloids showing no cytotoxic activity against tumor cells [15]. In conclusion, *L. radiata* extracts can act synergistically to reduce toxicity and expand pharmacological effects, making them suitable for agricultural or topical use, while purified compounds may be applied in targeted medical therapies.

### 3.7. Wound Healing Ability of L. radiata Leaf Extracts Obtained Through VUAE

**Figure 5:**
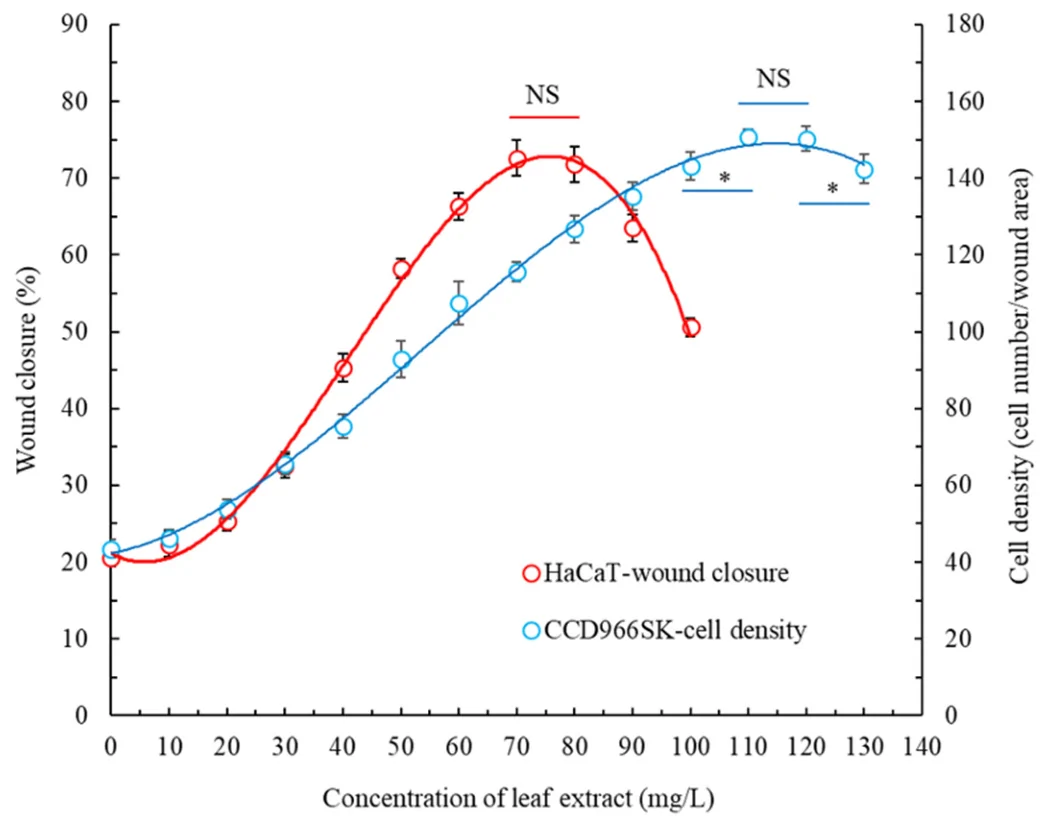
Wound healing abilities of *L. radiata* leaf extracts obtained through VUAE. Extraction conditions: extraction solvent, 80% ethanol; liquid-to-solid (L/S) ratio, 20 mL/g; extraction temperature, 40 °C; extraction time, 30 min; ultrasonic power, 240 W; and vacuum pressure, −0.05 MPa. Data are presented as the means ± standard deviations of 40 wounded cells. (NS, nonsignificant; *p* > 0.05; significance, * *p* < 0.05).

The leaf extract demonstrated strong antioxidant and anti-inflammatory properties (Figure 3), while the bulb extract displayed significant cytotoxicity (Figure 4). Considering the various stages of wound healing—hemostasis, inflammation, proliferation, and tissue remodeling [46]—we tested the leaf extract’s effect on cell models. Figure 5 shows that increasing the concentration of the leaf extract led to an initial rise in cell density at the CCD966SK wound site, which then leveled off, with the optimal concentration being 110 mg/L, where the cell density reached 150.6 ± 2.2 cells/wound area. For epidermal wound healing, a different trend was observed: the healing effect on HaCaT wounds improved with increasing leaf extract concentration up to a point, then declined, with the best result at 70 mg/L, achieving a wound healing capability of 72.6% ± 2.3%. An optimal concentration of 80 mg/L provided a balanced efficacy for both cell types. Compared to the positive control allantoin, the wound healing capacity in HaCaT and CCD966SK cells was 95.1% ± 7.6% and 167.2 ± 10.3 cells/wound area, respectively, indicating that the leaf extract possesses significant wound healing properties. This effect may be attributed to its alkaloids and phenolic compounds, particularly flavonoids [47]. However, since the optimal concentration for wound healing is lower than that for other pharmacological effects, and excessively high concentrations may impair wound healing in HaCaT cells, we recommend using the leaf extract alone to maximize its therapeutic benefits in wound healing. While previous studies have mainly focused on the activity of individual compounds [46,48], this research is the first to demonstrate the wound-healing potential of *L. radiata* extract.

### 3.8. Chemical Composition of L. radiata Leaf Extracts Obtained Through VUAE

Given that the leaf extract exhibited the most promising multifunctional bioactivities and the lowest cytotoxicity among the three plant parts studied, its detailed chemical composition was further analyzed to understand the synergy underlying its pharmacological properties. Studies indicate that the primary active compounds in *L. radiata* leaf extract are primarily AAs and polyphenols [2,3]. To gain deeper insight into the potential pharmacological mechanisms underlying the various physiological effects of *L. radiata* leaf extract, these two groups of compounds were specifically analyzed. Table 5 presents the major AA composition and their relative content in *L. radiata* leaf extract obtained through VUAE. Components with less than 0.5% relative content were omitted, resulting in the identification of 31 compounds. Among these, those exceeding 5% relative content, listed from highest to lowest, were: lycorine (10.23%), galanthamine (8.17%), hippeastrine (8.02%), lycoramine (6.06%), oduline (6.04%), pseudolycorine (5.93%), and tazettine (5.22%). In contrast, the bulb extract contained tazettine as the most abundant compound, followed by homolycorine, lycorenine, and galanthamine [18], which may explain the differing pharmacological effects between bulb and leaf extracts. Moreover, nine compounds found in the leaf extract—such as dihydro-latifaliumin C, *N*-demethyl-galanthamine, lycorine, lycoramine, galanthamine, pluviine, oduline, vittatine, and crinamidine—were also detected in the bulb extracts of *L. aurea*, *L. radiata*, and *L. guangxiensis* obtained using 90% ethanol combined with UAE [16]. Additionally, dihydrolycorine, galanthamine *N*-oxide, 8-demethyl-homolycorine-α-*N*-oxide, radiatine, and narciclasine were identified in the 95% ethanol extracts of *L. radiata* bulbs [14]. Clearly, *L. radiata* leaf extracts prepared by VUAE contain a wide variety of alkaloids, contributing to their diverse pharmacological properties. Regarding the AAs type, the leaf extract comprises seven classes, with the following relative proportions: homolycorine-type (26.91%), lycorine-type (24.55%), galanthamine-type (21.47%), crinine-type (12.00%), tazettine-type (7.00%), haemanthamine-type (4.09%), and narciclasine-type (3.57%). This profile differs markedly from bulb extracts, which are predominantly composed of galanthamine-type alkaloids, accounting for 40% to 65% of the total [16].

Table 6 presents the types of polyphenols and their relative contents detected in the leaf extract. A total of eighteen polyphenols were identified. Those with relative contents exceeding 5%, ranked from highest to lowest, included rutin (21.63%), quercetin (16.71%), cinnamic acid (12.43%), gallic acid (9.21%), caffeic acid (7.15%), and kaempferol (6.57%). Flavonoids were the predominant group, comprising 60.39% of the total polyphenols, with the remainder being phenolic acids. Additionally, cinnamic acid, caffeic acid, and *p*-coumaric acid were also found in *L. radiata* plants extracted with methanol using UAE [49]. Interestingly, flavonoids generally exist predominantly as glycoside derivatives; however, in *L. radiata* leaf extract, they are found in higher concentrations as aglycones. This phenomenon may result from rapid hydrolysis triggered by highly active endogenous β-glucosidases in L. radiata leaves. Alternatively, the leaves might modulate downstream metabolic enzymes in response to environmental defense requirements, favoring free aglycones as the primary accumulated form. Future studies could employ high-temperature rapid enzyme quenching techniques to determine whether this observation reflects the plant’s actual physiological state or is an artifact of enzymatic hydrolysis during the extraction process.

After identifying the active ingredients in these leaf extracts, we conducted an initial evaluation of their pharmacological sources by calculating the binding affinities between these active compounds and enzymes using the AutoDock Vina molecular docking technique. Among the leaf extracts, rutin, epicatechin gallate, narciclasine, and quercetin showed the strongest binding to tyrosinase, with binding energies of −9.23 kcal/mol, −8.81 kcal/mol, −8.73 kcal/mol, and −8.56 kcal/mol, respectively, which may account for skin-whitening effects. The ability of these polyphenolic compounds to inhibit tyrosinase activity is well established [50,51,52], whereas the mechanism by which narciclasine inhibits melanin production has only recently been clarified [53]. The highest binding affinities of rutin, narciclasine, luteolin, (+)-5,6-dehydrolycorine, and apigenin for the COX-1 and COX-2 enzymes ranged from −9.52 to −11.37 kcal/mol, −8.87 to −10.25 kcal/mol, −8.83 to −9.86 kcal/mol, −8.51 to −9.64 kcal/mol, and −8.53 to −9.52 kcal/mol, respectively, suggesting these compounds may contribute to the anti-inflammatory effects. Rutin, narciclasine, luteolin, and apigenin have long been recognized as effective anti-inflammatory agents [49,53,54,55], whereas the anti-inflammatory potential of (+)-5,6-dehydrolycorine remains to be established. Concerning anti-aging effects, the highest binding affinities of rutin, epicatechin gallate, luteolin, narciclasine, and haemanthamine for four enzymes related to aging ranged from −9.25 to −10.84 kcal/mol, −8.86 to −9.63 kcal/mol, −8.63 to −9.55 kcal/mol, −8.37 to −9.24 kcal/mol, and −8.28 to −8.84 kcal/mol, respectively. Rutin, epicatechin gallate, and luteolin have demonstrated significant antiaging properties [56,57,58]. The anti-aging effects of narciclasine have only recently been identified, mainly in preventing vascular aging [59], while the antiaging activity of haemanthamine has yet to be confirmed. Lastly, the highest binding affinities of kaempferol-*O*-rutinoside, rutin, quercetin, and narciclasine for key microbial enzyme targets—tyrosyl-tRNA synthetase and sterol 14α-demethylase—ranged from −9.20 to −10.52 kcal/mol, −9.07 to −10.25 kcal/mol, −8.87 to −9.65 kcal/mol, and −8.05 to −9.42 kcal/mol, respectively, which may explain their antimicrobial effects. These compounds have demonstrated broad-spectrum antibacterial activity and effective inhibition of fungal growth [60,61,62]. Nonetheless, further pharmacological studies on the isolated compounds are necessary to validate these mechanisms.

### 3.9. Transdermal Absorption and Permeation of Leaf Extracts and Lotions

Drawing on previous research and the required concentrations for the leaf extract to deliver its pharmacological benefits—such as skin whitening, anti-inflammatory, anti-aging, and antimicrobial effects—this study utilized a concentration of 400 mg/L. For wound-healing purposes, a concentration of 80 mg/L was applied. Given that the leaf extract contains many large flavonoid and alkaloid molecules, lotions containing the extract were also examined for transdermal absorption to determine if interactions between the extract’s compounds and lotion ingredients influenced skin absorption. The results showed that low-concentration extracts (80 mg/L) took longer to penetrate cumulatively, requiring 8–10 h to reach a stable equilibrium. In contrast, high-concentration extracts (400 mg/L) penetrated faster, stabilizing within about 6–8 h. The transdermal penetration efficiency of the leaf extracts across the Strat-M^®^ membrane ranged from 91.31% to 92.56%, whereas lotions containing the leaf extracts had lower efficiency, ranging from 56.34% to 65.39% (Table 7). For example, at 400 mg/L, the total transdermal absorption and penetration efficiency ranged from 78.98% to 97.54% (combining 6.23% retention and 91.31% penetration). Lower concentrations resulted in slower cumulative penetration but better overall absorption, while higher concentrations showed the opposite trend. These outcomes were primarily influenced by the concentration gradient and saturation effects [63]. Additionally, the lotion base significantly reduced penetration efficiency by about 15–20%. Future research could explore using liposome encapsulation in lotions to enhance penetration and absorption efficiency.

## 4. Conclusions

This study establishes *L. radiata* as a promising multifunctional bioresource, especially when extracted using the VUAE technique. The VUAE method achieved the highest extraction yields and increased levels of phenolics and flavonoids, resulting in superior pharmacological effects—particularly in leaf extracts. These leaf extracts exhibited strong antioxidant, skin-whitening, anti-inflammatory, wound-healing, and anti-aging properties, while bulb extracts showed significant antimicrobial effects. All extracts maintained cell viability above 70%, supporting a favorable safety profile. Chemical analysis revealed a wide variety of alkaloids and polyphenols that act synergistically, helping to reduce the toxicity often seen with isolated pure compounds. The high skin penetration ability of the leaf extracts highlights their suitability for topical use. In summary, VUAE enhances the bioactive qualities of *L. radiata*, with leaf extracts offering considerable promise for cosmetic, dermatological, and agricultural applications. Future studies should aim at developing formulations and conducting in vivo testing to support industrial use.

## Figures and Tables

**Table 1:** Antioxidant properties of *L. radiata* extracts obtained using different extraction techniques.

	TPC (mg GAE/g dw)			TFC (mg RE/g dw)		
	Leaf Extract	Bulb Extract	Root Extract	Leaf Extract	Bulb Extract	Root Extract
Maceration	93.1 ± 1.6	112.3 ± 2.5	86.7 ± 1.2	90.2 ± 2.8 ^b^	76.3 ± 2.7 ^a^	78.5 ± 1.5 ^a^
UAE	98.4 ± 2.2	146.8 ± 5.7	96.3 ± 3.8	105.6 ± 3.1 ^b^	80.6 ± 2.5 ^a^	81.2 ± 1.8 ^a^
VUAE	125.1 ± 3.4	174.1 ± 6.2	115.3 ± 5.4	130.4 ± 5.2 ^b^	94.9 ± 5.1 ^a^	98.7 ± 3.4 ^a^

In each row, different letters (a,b) indicate significant differences (*p* < 0.05).

**Table 2:** Whitening properties of *L. radiata extracts* prepared by VUAE (IC_50_, mg/L).

	Extracellular Tyrosinase Activity	Intracellular Tyrosinase Activity	Melanin Content in HEMn Cells
Leaf extract	93.5 ± 1.4	51.7 ± 1.6 ^a^	49.6 ± 2.2 ^a^
Bulb extract	131.4 ± 4.9	102.6 ± 3.4 ^a^	74.8 ± 3.1 ^b^
Root extract	501.8 ± 10.2 ^a^	493.5 ± 5.2 ^a^	486.7 ± 9.3 ^a^

In each row, different letters (a,b) indicate significant differences (*p* < 0.05).

**Table 3:** Anti-aging activities of *L. radiata* extracts obtained through VUAE (IC_50_, mg/L).

	Leaf Extract	Bulb Extract	Root Extract
MMP-1	61.2 ± 7.5 ^a^	76.5 ± 9.1 ^b^	58.3 ± 3.2 ^a^
Collagenase	225.7 ± 12.4 ^a^	247.3 ± 10.6 ^b^	218.6 ± 15.2 ^a^
Elastase	50.8 ± 3.4 ^a^	52.3 ± 2.7 ^b^	53.2 ± 3.8 ^b^
Hyaluronidase	146.8 ± 12.5 ^b^	151.7 ± 10.3 ^b^	137.2 ± 10.6 ^a^

In each row, different letters (a,b) indicate significant differences (*p* < 0.05).

**Table 4:** MBC and MFC of *L. radiata extracts* obtained through VUAE.

		Leaf Extract	Bulb Extract	Root Extract
Bacteria (MBC, mg/L)				
	*E. coli*	208 ± 31.2	125 ± 20.4	800 ± 81.6
	*P. aeruginosa*	292 ± 31.2	108 ± 11.8	916 ± 23.6
	*S. aureus*	350 ± 40.8	150 ± 40.8	1025 ± 20.4
	*C. acnes*	250 ± 40.8	125 ± 20.4	1250 ± 204.1
Fungi (MFC, mg/L)				
	*C. albicans*	558 ± 31.2	158 ± 11.8	– *
	*A. brasiliensis*	633 ± 47.1	225 ± 20.4	–

* >2000 mg/L.

**Table 5:** Major Amaryllidaceae alkaloid composition and relative content in *L. radiata* leaf extract obtained through VUAE.

Peak No.	Rt (min) *	Identification **	Alkaloid Type	Relative Content (%)
1	3.15	1,11-didehydrogalanthamine	galanthamine-type	0.54
2	3.53	11-hydroxyvittatine N-oxide	crinine-type	0.73
3	3.86	3-hydroxylatifaliumin C	tazettine-type	0.52
4	4.92	dihydro-latifaliumin C	tazettine-type	1.67
5	6.43	2α-Hydroxy-3-hydro-6-*O*-methyloduline	homolycorine-type	2.23
6	6.98	N-demethyl-galanthamine	galanthamine-type	2.16
7	9.75	crinamabine	crinine-type	0.68
8	12.78	lycorine	lycorine-type	10.23
9	13.06	dihydrolycorine	lycorine-type	4.61
10	14.02	pseudolycorine	lycorine-type	5.93
11	14.18	(+)-5,6-dehydrolycorine	lycorine-type	0.53
12	14.83	lycoramine	galanthamine-type	6.06
13	17.15	galanthamine	galanthamine-type	8.17
14	18.17	lycoramine N-oxide	galanthamine-type	0.62
15	21.36	galanthamine N-oxide	galanthamine-type	3.92
16	23.45	pluviine	lycorine-type	3.25
17	25.92	oduline	homolycorine-type	6.04
18	26.78	ambelline	crinine-type	2.56
19	27.31	vittatine	crinine-type	3.14
20	28.05	crinamidine	crinine-type	3.81
21	29.11	hippeastrine N-oxide	homolycorine-type	1.25
22	29.82	haemanthamine	crinine-type	1.08
23	30.95	hippeastrine	homolycorine-type	8.02
24	31.27	8-demethyl-homolycorine-α-N-oxide	homolycorine-type	3.71
25	31.83	2α-hydroxy-6-*O*-methyloduline	homolycorine-type	1.56
26	32.75	(+)-8,9-methylenedioxylhomolycorine N-oxide	homolycorine-type	0.62
27	33.08	9-*O*-demethylhomolycorine N-oxide	homolycorine-type	2.35
28	33.42	2-methoxyoduline	homolycorine-type	1.13
29	34.62	radiatine	haemanthamine-type	4.09
30	35.57	narciclasine	narciclasine-type	3.57
31	37.45	tazettine	tazettine-type	5.22

* Rt: retention time; ** Peaks 6, 8, 9, 10, 12, 13, 15, 16, 19, 22, 23, 30, and 31 were identified via LC-MS/MS by comparison with reference standards, whereas the remaining peaks were tentatively identified through comparison with literature data from Li et al. [14].

**Table 6:** Major polyphenolic compound composition and relative content of *L. radiata* leaf extract obtained through VUAE.

Rt (min) *	Chemical Compounds	Categories	Relative Content (%)
5.09	cinnamic acid	phenolic acid	12.43
5.46	gallic acid	phenolic acid	9.21
6.27	chlorogenic acid	phenolic acid	2.58
8.52	4-hydroxybenzoic acid	phenolic acid	2.07
10.52	caffeic acid	phenolic acid	7.15
10.68	*p*-coumaric acid	phenolic acid	3.25
12.15	ferulic acid	phenolic acid	2.41
15.31	sinapinic acid	phenolic acid	0.51
17.46	rutin	Flavonoids	21.63
19.85	catechin	Flavonoids	3.82
20.18	luteolin-7-*O*-β-D-glucoside	Flavonoids	1.05
21.63	epicatechin gallate	Flavonoids	4.42
23.15	kaempferol-3-*O*-glucoside	Flavonoids	0.54
23.57	luteolin	Flavonoids	1.18
24.05	quercetin	Flavonoids	16.71
25.47	kaempferol	Flavonoids	6.57
27.42	kaempferol-*O*-rutinoside	Flavonoids	0.75
32.68	apigenin	Flavonoids	3.72

* Rt: retention time.

**Table 7:** In vitro penetration of *L. radiata* leaf extract across a Strat-M membrane using static Franz-type diffusion cells.

		Amount Recovery (% of Applied Dose)			
		Donor	Strat-M^®^ Membrane	Receptor	Total
400 mg/L	Extracts	2.14	6.23	91.31	99.67
	Lotion	20.83	22.64	56.34	99.81
80 mg/L	Extracts	1.96	5.41	92.56	99.93
	Lotion	16.35	18.23	65.39	99.97

## Data Availability

All data generated or analyzed during this study are included in this published article.
